# West Nile Virus Infection in Killer Whale, Texas, USA, 2007

**DOI:** 10.3201/eid1708.101979

**Published:** 2011-08

**Authors:** Judy St. Leger, Guang Wu, Mark Anderson, Les Dalton, Erika Nilson, David Wang

**Affiliations:** Author affiliations: SeaWorld, San Diego, California, USA (J. St. Leger, E. Nilson);; Washington University School of Medicine, St. Louis, Missouri, USA (G. Wu, D. Wang);; University of California at Davis, Davis, California, USA (M. Anderson);; SeaWorld, San Antonio, Texas, USA (L. Dalton)

**Keywords:** West Nile virus, killer whale, Orcinus orca, cetaceans, nonsuppurative encephalitis, zoonoses, arboviruses, viruses, dispatch

## Abstract

In 2007, nonsuppurative encephalitis was identified in a killer whale at a Texas, USA, marine park. Panviral DNA microarray of brain tissue suggested West Nile virus (WNV); WNV was confirmed by reverse transcription PCR and sequencing. Immunohistochemistry demonstrated WNV antigen within neurons. WNV should be considered in cases of encephalitis in cetaceans.

West Nile virus (WNV) is a single-stranded RNA virus of the genus *Flavivirus* that is transmitted by mosquitoes. In humans and animals, WNV has been associated with a spectrum of clinical conditions from asymptomatic infections to sudden death. These have been identified in a variety of animal species. Among marine mammals, WNV infection has been reported in a harbor seal (*Phoca vitulina*) (1). We describe WNV infection in a killer whale (*Orcinus orca*) and seroprevalence in conspecific cohort and noncohort groups.

## The Study

In 2007, a 14-year-old male killer whale at a marine park in San Antonio, Texas, USA, died suddenly without notable premonitory signs. On gross examination, mild multifocal meningeal hyperemia and petechial parenchymal hemorrhage were noted in the right cerebrum and cerebellum. The left hemisphere of the brain appeared normal. Focally extensive tan discoloration and fibrosis were present in the right accessory lung lobe with associated hemorrhage and congestion. Both lung lobes were mildly and diffusely heavy and wet. All thoracic and abdominal lymph nodes were moderately enlarged and edematous. The second gastric chamber displayed numerous chronic and active ulcerations of 1.5–2 cm. Fresh and buffered 10% formalin-fixed specimens were collected. Fresh tissues were stored at –80°C. Tissues fixed in 10% buffered formalin were processed routinely and stained with hematoxylin and eosin for histologic examination.

Histologic review demonstrated moderate multifocal subacute vasculitis and nonsuppurative encephalitis. Inflammatory lesions of the central nervous system were focused in gray matter of the medulla oblongata, pons, mesencephalan, and cerebellum. Lesions were bilateral but more severe on the right side. Meninges demonstrated moderate focally extensive and multifocal areas of acute meningeal congestion and hemorrhage. Mild multifocal lymphocytic infiltrates expanded the leptomeninges. Blood vessels demonstrated mild to moderate acute necrosis and lymphocytic and contained plasmacytic and neutrophilic infiltrates within vascular walls. Encephalitis was characterized by perivascular lymphocytes and fewer plasma cells expanding the Virchow-Robbins spaces. Small, scattered, perivascular ring hemorrhages were noted. A few multifocal loosely arranged glial nodules were within cerebral white matter.

Predominant lesions in the lungs were areas of chronic and active abscessation amid a focally extensive area of mixed inflammation and fibrosis. There was moderate diffuse acute pulmonary edema and congestion. Gastric ulcerations were present in the first gastric chamber and were chronic and active. They were characterized by central ulcerations with necrosis and a mixed inflammatory infiltrate surrounded by variable fibrosis and a rim of epithelial hyperplasia. Changes in spleen, lymph node, and kidney included acute edema, congestion, and vascular dilation.

Conventional diagnostic assays were performed for aerobic, anaerobic, and fungal microbes in liver, lung, kidney, cerebrospinal fluid, and brain. All yielded minimal growth of *Escherichia coli*.

The final diagnosis was fulminant peracute bacteremia and septicemia secondary to a primary viral infection associated with nonsuppurative encephalitis. Published etiologic considerations for cetacean nonsuppurative encephalitis include morbillivirus and protozoal infections (*2*). A DNA microarray with highly conserved sequences from >1,000 viruses was selected to screen for known and novel viruses (*3*). Total RNA was extracted from brain tissue and hybridized to a microarray as described (*4*). Analysis of the resulting hybridization pattern demonstrated a strong hybridization signal to many oligonucleotide probes on the microarray from the family *Flaviviridae*, in particular to WNV. Consensus reverse transcription PCR primers (*5*) targeting WNV were used to confirm the microarray results. Sequencing of the 261-bp amplicon (GenBank accession no. HQ610502) yielded a sequence with 99% nt identity and 100% aa acid identity to WNV strain OK03 (GenBank accession no. EU155484.1), a strain originally identified in Oklahoma, USA.

To further support a WNV diagnosis, we performed immunohistochemical staining on brain tissue. The immunoperoxidase stain used was a commercial rabbit polyclonal antibody (BioRelience Corp., Rockville, MD, USA) with peroxidase-tagged goat antirabbit immunoglobulin G (DakoCytomation, Carpinteria, CA, USA) bridge and 3-amino-9-ethylcarbazole (DakoCytomation) as the chromogen. This staining demonstrated abundant WNV antigen within the cytoplasm of a small number of neurons and glial cells and in fewer macrophages in the brain tissue (Figure).

**Figure:**
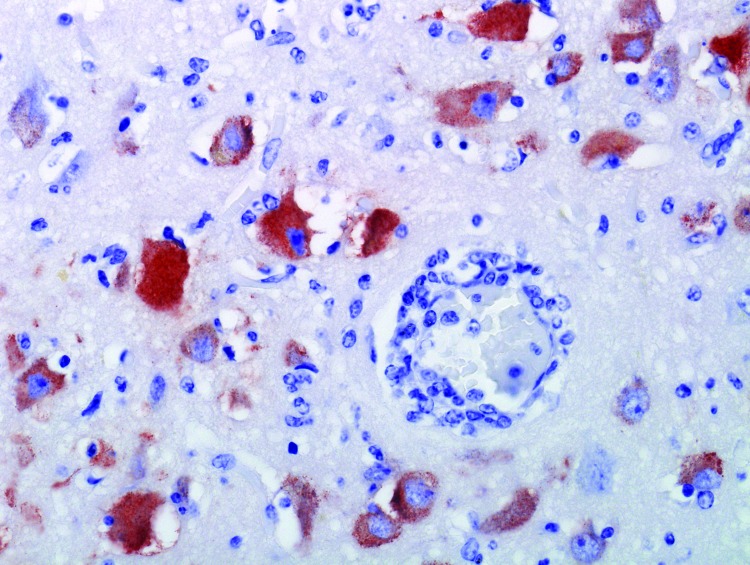
Brain specimen from killer whale (*Orcinus orca*) with West Nile virus infection that died at a marine park, San Antonio, Texas, USA, 2007. Neurons and glial cells demonstrate abundant intracytoplasmic West Nile viras antigen. Blood vessel demonstrates mild vasculitis and perivascular lymphocytic infiltrate. Original magnification ×200.

We evaluated WNV exposure within the same cohort, as well as a geographically distant cohort of whales by using serologic testing. All testing was performed at the same laboratory by using a standard plaque-reduction neutralization test. In this assay, a 90% neutralization cutoff was used (*6*). A 90% plaque-reduction titer >10 was considered positive. Serum from the affected whale and 5 cohort killer whales from the same marine park in San Antonio as well as 5 whales housed at another facility in Orlando, Florida, USA, were evaluated. In each facility, the animals have regular contact with each other. The facilities are geographically separated so the animals do not have exposure to those in the other park. All 6 animals from Texas had 90% plaque-reduction titers >10, ranging from 40 to 80. The 5 whales housed together in Orlando had no measurable titer.

## Conclusions

We demonstrate that WNV can infect and cause disease in killer whales. These findings broaden the known host tropism of WNV to include cetaceans in addition to previously known pinnipeds. Although we cannot definitively attribute the cause of death of this whale to WNV, the observed lesions are consistent with those caused by WNV in other animals. The serologic results demonstrate that subclinical infections can occur and that exposure can be variable. We did not determine specific dates of exposure for these populations. Both Bexar County, Texas, and Orange County, Florida, have had WNV in wildlife since 2002. We continue annual serology on previously negative animals to document seroconversion. Mosquito management practices are similar in both facilities and have been expanded since this diagnosis. Differences in WNV prevalence or mosquito numbers may have played a role in the different serologic results.

Health evaluations of free-ranging and captive cetaceans should include WNV serology to assess exposure rates. This report focuses on killer whales, but the “loafing” behavior (stationary positioning at the water’s surface) is commonly seen in many coastal dolphins, thereby increasing the likelihood of mosquito bites and exposure to WNV. Serologic screening of bottlenose dolphins (*Tursiops truncatus*) from the Indian River Lagoon demonstrated WNV titers (*7*). WNV-associated disease in these animals has not been reported. Active screening for WNV may enhance diagnostic investigations.

As with many species of birds and mammals, WNV infection carries a risk for zoonotic transmission. Until the implications of this infection in marine mammals are better understood, biologists and veterinarians working with cetaceans should consider this possibility. Potential viral shedding can occur through the oropharygeal cavity and feces as well as through blood and organs during necropsies.

Finally, our study demonstrates the broad applicability of using panviral microarray-based diagnostics. Even though PCR diagnostics are well developed for WNV, the agent was not initially considered as a potential pathogen in this species. Panviral microarray can be used not only to identify novel viruses but also to detect unsuspected agents.
